# Follistatin like-1 (Fstl1) is required for the normal formation of lung airway and vascular smooth muscle at birth

**DOI:** 10.1371/journal.pone.0177899

**Published:** 2017-06-02

**Authors:** Xue Liu, Yingying Liu, Xiaohe Li, Jing Zhao, Yan Geng, Wen Ning

**Affiliations:** 1 State Key Laboratory of Medicinal Chemical Biology, College of Life Sciences, Nankai University, Tianjin, China; 2 Model Animal Research Center, Nanjing University, Nanjing, China; 3 School of Pharmaceutical Science, Jiangnan University, Wuxi, Jiangsu, China; Children's Hospital of Los Angeles, UNITED STATES

## Abstract

Fstl1, a secreted protein of the BMP antagonist class, has been implicated in the regulation of lung development and alveolar maturation. Here we generated a *Fstl1-lacZ* reporter mouse line as well as a *Fstl1* knockout allele. We localized *Fstl1* transcript in lung smooth muscle cells and identified Fstl1 as essential regulator of lung smooth muscle formation. Deletion of *Fstl1* in mice led to postnatal death as a result of respiratory failure due to multiple defects in lung development. Analysis of the mutant phenotype showed impaired airway smooth muscle (SM) manifested as smaller SM line in trachea and discontinued SM surrounding bronchi, which were associated with decreased transcriptional factors myocardin/serum response factor (SRF) and impaired differentiation of SM cells. *Fstl1* knockout mice also displayed abnormal vasculature SM manifested as hyperplasia SM in pulmonary artery. This study indicates a pivotal role for Fstl1 in early stage of lung airway smooth muscle development.

## Introduction

Our lung is optimized for oxygen supply of organism. To facilitate gas exchange, the lung develops two highly branched and tree-like systems, the airways and the vasculature, to conduct air and blood supply. Both systems contain a intertwined component of smooth muscle (SM)—airway SM (ASM) controls the diameter of airway tubes, vascular SM (VSM), blood vessels. While SM is crucial for normal respiratory function, disruption of lung SM development and function in humans can lead to chronic healthy problems, such as overproliferation observed in asthma and hypertrophy associated with pulmonary hypertension [1].

SM in the lung is thought to derive from the multipotent progenitors in developing lung mesoderm. The lung is a highly complex organ that develops from the ventral foregut via reciprocal interactions between foregut endoderm and surrounding mesoderm [2]. In addition to the important source of signals for epithelial growth and differentiation and branching morphogenesis, the mesoderm is also a source of multi-potential progenitors giving rise to a variety of cell lineages, including ASM and VSM [3]. As development proceeds, those progenitors committed to become airway SM move proximally and envelop the airway tubes, by contrast, vascular SM become incorporated into vessels several days later [4]. Several key signals, including SHH, BMPs, WNTs, VEGF, PDGF, FGFs, TGF-β and RA, that regulate reciprocal interactions between foregut endoderm and surrounding mesoderm, may also coordinate the various specification and differentiation processes occurring during SM development [1,5]. However, the mechanisms underlying lung mesodermal development, in particular SM development, are largely unknown [1,3,4].

Follistain-like 1 (Fstl1) is a member of the secreted protein acidic rich in cysteine (SPARC) family and has been implicated in many different signaling pathways, including BMP and TGF-β signaling [6]. In multiple biological and pathological processes, such as immunomodulation [7–9], tumorigenesis [10–12], vascularization [13–15], fibrogenesis [16] and embryonic development [17–20], Fstl1 has shown diverse and cell type-specific functions, including the regulation of cell proliferation, apoptosis, differentiation, and migration. In lung development, loss-of-function experiments using null mice have unveiled Fstl1 as a BMP antagonist in regulating alveolar epithelial differentiation/maturation [17]. In addition, Fstl1 has been reported to be ubiquitously expressed in embryonic lung with high levels in mesenchyme surrounding airways and walls of blood vessels [21]. The dynamic spatial expression pattern suggests another potential role of Fstl1 in the development of mesenchymal-derived lineages in the lung, specially ASM and VSM.

In this study, we generated a *Fstl1-LacZ* reporter mouse line as well as *Fstl1* knockout mouse line to detect the spatiotemporal expression pattern of Fstl1 and to examine the role of Fstl1 in lung SM development. Homozygous mice of both strains died at birth due to respiratory distress. In addition to the reported defects in lung development [17], we showed that Fstl1 deficiency leads to a severe airway SM defect in *Fstl1* null embryos. We localized *Fstl1* transcript in lung SM cells and found that Fstl1 is essential for ASM formation via regulating the critical transcriptional factors myocardin and SRF. Thus, Fstl1 plays an important role in the lung mesenchyme by regulating ASM development.

## Materials and methods

### Ethics statement

All mice were housed and cared for in a specific-pathogen free (SPF) facility at Nankai University. All experimental procedures were approved by the Institutional Animal Care of Experimental Animal Center, Nankai University. Pregnant mice were sacrificed by cervical dislocation, the embryos were isolated quickly after uterus was removed. After decapitation of the embryos, the specimens were rapidly harvested and used for subsequent experiments. All efforts were made to minimize animal suffering and all approaches were used to minimize the number of animal used.

### Mouse strains

To generate *Fstl1-LacZ* reporter mice, we inserted an *ECMV IRES-LacZ-poly(A)* cassette and a *β-actin promoter-neo-SV40 pA* cassette into the intron between exon 2 and exon 3. The two cassettes were divided by a *loxP* site and flanked by two *FRT* sites. The exon 3 and 4 were flanked by two *loxP* sites in the same orientation with the first one. Due to the *LacZ* cassette followed by poly(A), Fstl1 expression can be labeled by X-gal staining in heterozygous *Fstl1-LacZ* mice. Southern blot was used to analysis the positive clones and WT mouse of 129 backgroud was used as negative control (S1A Fig). *Fstl1*^*flox/+*^ mice were generated by crossing *Fstl1-LacZ* mice with *ACTB*-*FLP* mice (*B6*.*SJL-Tg(ACTFLPe)9205Dym/JNju*, purchased from Model Animal Research Center of Nanjing University). After crossing *Fstl1*^*flox/+*^ with *EIIa-Cre* mice (*B6*.*FVB-Tg(EIIa-cre)C5379Lmgd/J*,*003724*, purchased from Model Animal Research Center of Nanjing University), we got heterozygous *Fstl1* exon 3 and 4 knockout mice strain (*Fstl1*^*+/−*^). Intercrossing *Fstl1*^*+/−*^ mice produced *Fstl1* exon 3 and 4 deleted mice (*Fstl1*^*−/−*^). Polymerase chain reaction (PCR) was performed to determine the genotypes of pups (S1B Fig) and Semi-qRT-PCR was used to detect the transcriptional levels of *Fstl1* exons (S1C Fig). *Fstl1*^*flox/+*^ mice were bred in C57BL/6J background and *Fstl1*^*+/−*^ mice were kept at C57BL/6J and mixed FVB/NJ and C57BL/6J background. Sequences of the specfic genotyping primer sets are as follows: *Fstl1-LacZ*, forward, 5’- AGCAGCGTTGTTGCAGTGCACGGC -3', reverse, 5'- TGCTACCTGCGCCAGCTGGCAGTTC -3', *Fstl1*^*flox/+*^, forward, 5’- ACATGGTG ACCATCCTTCGG -3’, reverse, 5’- TTCTAGGTTCCTCCTAAAAC -3’, *Fstl1*^*+/−*^, forward, 5’- CACATGGTGACCATCCTTCGG -3’, reverse, 5’- GCTGCTTCA CTTAATCTCCTGCTGT -3’ and 5’- TTGTGTTGTCCTTGGTTGTTTTCA -3’. The exon 2 knockout mice line was described before [17]. All mice were maintained in an SPF facility in Experimental Animal Center of College of Life Sciences, Nankai University.

### X-gal staining

X-gal staining was performed as previously reported [22]. In brief, embryonic lungs were isolated and fixed in 0.2% glutaraldehyde (in 0.1 M NaPO_4_ buffer) for 1–2 hours at 4°C. Then the lungs were washed 30 minutes in rinse solution (2 mM MgCl_2_, 0.2% NP-40, 0.1% sodium deoxycholate) for 3 times and incubated in X-gal staining solution (1 mM X-gal, 5 mM K_4_Fe(CN)_6_, 5 mM K_3_Fe(CN)_6_, 2 mM MgCl_2_ in rinse solution) overnight shaking in the dark at 37°C. Then, the X-gal-stained lungs were rinsed in PBS and post-fixed with 10% neutral formalin. Lastly, the samples were rinsed with PBS and used for whole-mount analysis and further processing for sectioning.

### Semi-qRT-PCR and qRT-PCR analysis

We performed RNA isolation and qRT-PCR analysis as previously described [17]. In brief, total RNA was isolated with Trizol (Invitrogen, Carlsbad, CA), cleaned with the RNeasy Mini Kit (Qiagen, Valencia, CA) and the DNA-free kit (Ambion, Austin, TX). cDNA was synthesized with the First Strand cDNA Synthesis Kit for RT-PCR according to the manufacturer’s protocols (Roche Diagnostics GmbH, Mannheim Germany), and Semi-qRT-PCR for each gene was performed in a 20 μl reaction system containing 1 μl cDNA, a final concentration of 1 μmol/L of each primer, and FastStart High Fidelity PCR System according to the manufacturer’s protocols (Roche Diagnostics GmbH, Mannheim Germany). qRT-PCR was performed by using SYBR GreenER qPCR SuperMix Universal (Invitrogen) according to the manufacturer’s protocols. Gene expression was measured relative to the endogenous reference gene, *β-actin*. To determine the transcriptional levels of different exons in *Fst1l*^*−/−*^ mice, we designed primers in different exons of *Fstl1* gene. Sequences of the specific primer sets are as follows: *β-actin*, forward, 5’- CCTCGCCTTTGCCGATCCGGATCTT -3’, reverse, 5’- CATGAGGTAGTCAGTC -3’, *Exon1-2* (*E1-2*), forward, 5’- TTCCTC GGAGCCTGGTGATAAGCGA -3’, reverse, 5’- AAGCTTGGCGATGGTCACCAG CGAGA -3’, *Exon3-4* (*E3-4*), forward, 5’- GAGGAACCTAGAAGCAAATC -3’, reverse, 5’- GTGCCCATCATAATCAACCT -3’, *Exon5-6* (*E5-6*), forward, 5’- AAAAGAAGTCTGCGAGTCCA -3’, reverse, 5’- CTTAAAGTACTTGTCTAGG ATCTC -3’, *Exon7-10* (*E7-10*), forward, 5’- AGCTTTGATAATGGCGACTC -3’, reverse, 5’- GGACATATCCTGTCTTCTCC -3’, *Fstl1*, forward, 5’- TTATGATGGG CACTGCAAAGAA -3’, reverse, 5’- ACTGCCTTTAGAGAACCAGCC -3’, *α-SMA*, forward, 5’- GCTGGTGATGATGCTCCCA -3’, reverse, 5’- GCCCATTC CAACCATTACTCC -3’, *myocardin*, forward, 5’- TCAATGAGAAGATCG CTCTCCG -3’, reverse, 5’- GTCATCCTCAAAGGCGAATGC -3’, *SRF*, forward, 5’- CCAGGTGTCGGAATCTGACAG -3’, reverse, 5’- GCTGACTTGCATG GTGGTAGA -3’.

### Alcian blue staining of embryonic tracheas

Alcian blue staining of embryonic tracheas was performed as previously reported [23]. Briefly, tracheas of E18.5 embryos were removed under stereoscope and fixed in 95% ethanol for 1 day. After fixation, the samples were degreased in 100% acetone for 1 day. Then the samples were fixed in 95% ethanol again for about 1 day and stained in Alcian blue solution (0.08% Alcian blue: glacial acetic acid: 100% ethanol = 1:1:10) at 37°C for 1–3 days. After staining, the samples were placed in 0.1% KOH until the soft tissue appeared transparent and then transferred to 100% glycerol via a graded series of glycerol in 0.1% KOH (25%, 50%, and 75%).

### Western blotting

The protein was extracted from tissue following standard protocols as described previously [17]. Briefly, total protein was extracted from E15.5 mice embryos, and equal amounts of protein were separated by SDS-PAGE and transferred to polyvinylidene difluoride membrane (PVDF: Roche Diagnostics GmbH, Mannheim Germany). After blocking with 5% non-fat milk, the membrane was incubated with goat anti-Fstl1 (R&D system, AF1738, 1:500) over night at 4°C. After reacting with horseradish peroxidase-conjugated secondary antibody, the signalings were detected by SuperSignal West Pico Chemiluminescent Substrate (Thermo Scientific, Rockford, U.S.A).

### Histology and immunohistology

Immunohistochemistry and immunofluorescence were performed as described previously [16]. In brief, for histological staining, mouse embryos were fixed in 10% neutral formalin, paraffin-embedded, sectioned, and stained with hematoxylin-eosin. Immunohistochemistry was performed on paraffin sections following standard protocols. Antibody used was monoclonal anti-α-Smooth Muscle actin-Cy3 (sigma-aldrich, clone 1A4, 1:200).

### Whole mount α-SMA staining for E18.5 tracheas

The whole mount staining protocol was kindly provided by Prof. Nan Tang. Briefly, dissected tracheas were fixed in DENT’s solution (Methanol: DMSO = 4:1) and washed in Methanol. Then the tissues were incubated in 5% H_2_O_2_ (DENT’s solution: 30% H_2_O_2_ = 5:1) and rehydrated through a graded series of methanol and finally washed in PBST. The tracheas were incubated in blocking buffer (5% Rabbit serum/ 3% BSA/ PBS/ 0.5% TritonX-100) and then in anti-α-SMA-Cy3 antibody over night at 4°C. The tracheas were washed in PBST and postfixed with 4% PFA and then dehydrated through a graded series of methanol. Finally the tracheas were cleared in BABB (benzyle alcohol: benzyle benzoate = 1:2) and analysed from the dorsal side.

### Mouse embryonic fibroblasts (MEFs) isolation and culture

The pregnant mice were sacrificed on day 13 of pregnancy by cervical dislocation, the uterine horns were dissected out and placed into a dish containing PBS. Each embryo was separated from its placenta and surrounding membranes. After the heads, tails, limbs, and most of the internal organs were removed, the embryos were minced and suspended in 1 ml of trypsin-EDTA and incubated in a dish at 37°C for 15 minutes, and then seeded into culture dishes in DMEM with 10% FBS. MEFs from WT and *Fstl1*^*−/−*^ were cultured to reach 60%-80% confluence and serum starved for 24 hours, and then treated with 5 ng/ml TGF-β1 (R&D Systems) for 12 hours. Cells were harvested with Trizol for gene expression analysis by qRT-PCR.

### Statistical analysis

Data were expressed as the mean ± SEM. Differences in measured variables between experimental and control group were assessed by using Student’s *t* tests. Results were considered statistically significant at p < 0.05. SPSS was used for statistical analysis.

## Result

### Expression of Fstl1 in airway and vascular SM cells

**Fig 1:**
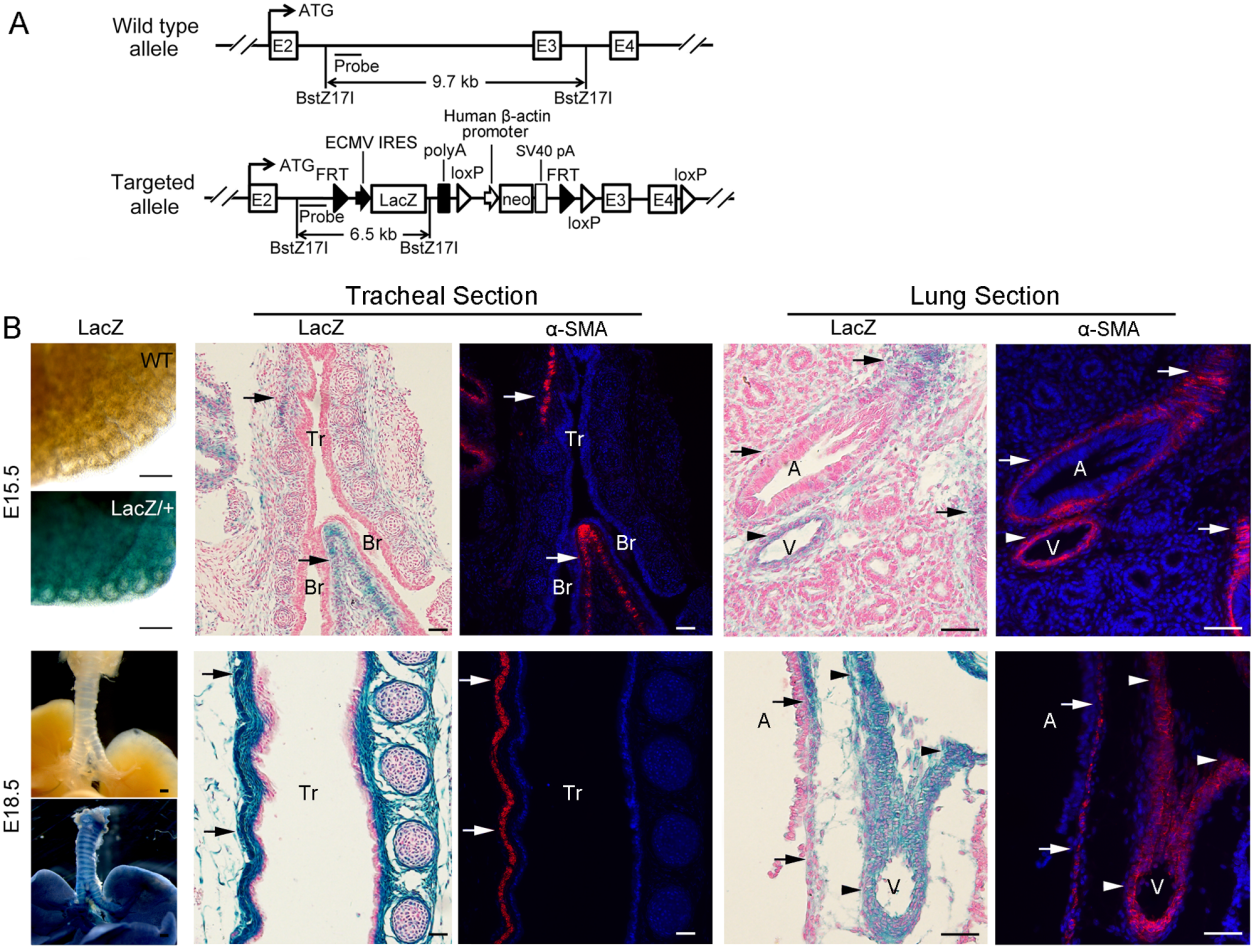
*Fstl1-LacZ* reporter mice confirmed Fstl1 expression in airway and vascular SM. (**A**) Schematic representation of generation of *Fstl1-LacZ* reporter mice. (**B**) Whole-mount X-gal staining of E15.5 and E18.5 WT and *Fstl1*^*LacZ/+*^ lungs and X-gal staining and α-SMA staining of the tracheal longitudinal sections and lung sections of E15.5 and E18.5 embryos. X-gal staining and α-SMA staining of *Fstl1*^*LacZ/+*^ adjacent trachea and lung sections showed co-staining of LacZ and α-SMA in airway (arrows) and blood vessel (arrowheads). Tr, trachea, Br, Bronchi, A, airway, V, blood vessel. Scale bars, whole-mount X-gal staining, 200 μm; X-gal staining and α-SMA staining of sections, 50 μm.

To localize *Fstl1* transcription with single cell resolution in the embryonic mouse lung, we generated a *Fstl1-LacZ* reporter mouse line by inserting a *FRT*-flanked *LacZ* cassette and a neomycin cassette in intron 2. In addition, three *loxP* sites were also inserted between the *LacZ* cassette and neomycin cassette, in intron 2 after *FRT* site, and in intron 4 respectively in the same orientation (Fig 1A). Fstl1 expression was stopped by *LacZ* sequence followed by poly(A) tail and the *Fstl1-lacZ* reporter allele predictably did not produce intact Fstl1 protein, which was confirmed by the postnatal death (S2A Fig) and abnormal formation of tracheal cartilage (S2B Fig) and lung atelectasis (S2C Fig) of homozygous *Fstl1-lacZ* (*Fstl1*^*LacZ/LacZ*^) reporter mice.

The heterozygous *Fstl1-LacZ* (*Fstl1*^*LacZ/+*^) mice allow us to visualize Fstl1 expression during embryogenesis *in vivo*. X-gal staining of lung sections showed a generalized expression of Fstl1 at E15.5 and E18.5, with high levels in lung mesenchyme surrounding airways (Fig 1B, arrows), as well as in walls of blood vessels (Fig 1B, arrowheads). Immunofluorescence staining of adjacent lung sections using antibodies specific for SM cells (α-SMA) exhibited the overlapping of LacZ-positive Fstl1 expressing cells with α-SMA-positive ASM cells (Fig 1B, arrows) and VSM cells (Fig 1B, arrowheads), confirming the localization of *Fstl1* transcripts in airway and vascular SM cells in embryonic lung.

### Generation of *Fstl1* knockout mice

**Fig 2:**
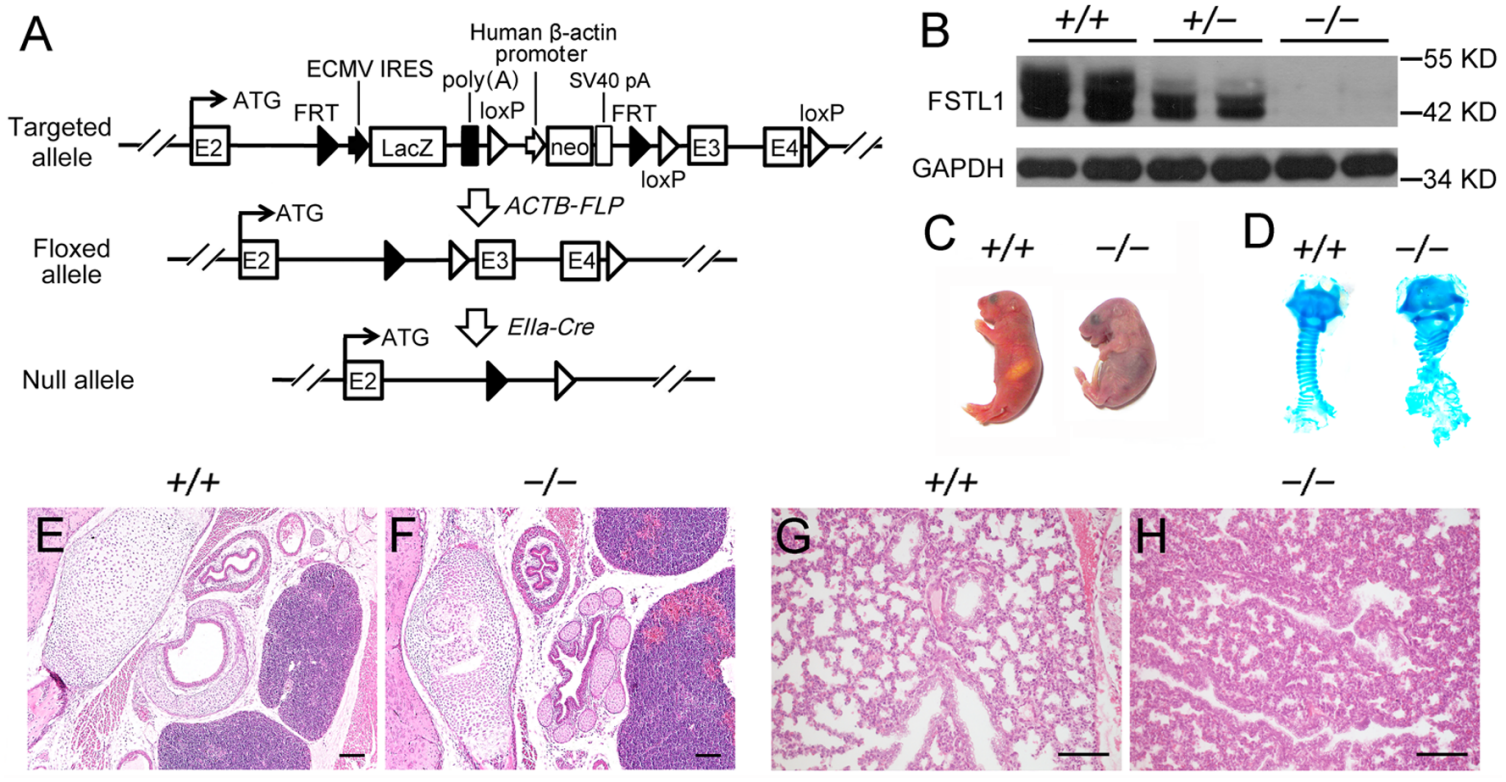
Generation of *Fstl1*^*−/−*^ mice. (**A**) Generation of *Fstl1* exon 3 and 4 floxed mice (*Fstl1*^*flox/+*^) by crossing *Fstl1-LacZ* with *ACTB-FLP* mice. *Fstl1*^*flox/+*^ mice were crossed with *EIIa-Cre* transgenic mice, resulting in exon 3 and 4 deletion. (**B**) Western blot analysis of Fstl1 protein from E15.5 embryos. (**C**) *Fstl1* knockout pups died of respiratory distress shortly after birth. (**D**) Alcian blue staining revealed impaired banding pattern of tracheal C-ring cartilage. H&E staining of E18.5 WT and *Fstl1*^*−/−*^ embryonic trachea (**E**, **F**) and lung (**G**, **H**) sections. Scale bars, 100 μm.

To determine the biological function of Fstl1 *in vivo*, we crossed *Fstl1*^*LacZ/+*^ mice with *ACTB-FLP* mice and then *EIIa-Cre* mice to generate *Fstl1*^*+/−*^ mice by deleting exon 3 and 4 which encode the first functional domain of Fstl1, FS domain [24,25] (Fig 2A). Removal of exon 3 and 4 should result in a frame shift in the coding region and western blot confirmed that exon 3 and 4 knockout allele did not produce intact Fstl1 protein (Fig 2B).

In both *Fstl1-LacZ* and *Fstl1*^*+/−*^ strains, their homozygous pups were born alive with multiple developmental malformations, and then died shortly after birth due to respiratory failure (S2A Fig and Fig 2C), findings consistent with the phenotypes observed in other *Fstl1* knockout strains [17–19]. *Fstl1*^*−/−*^ embryos showed greatly disorganized tracheal cartilage (Fig 2D, 2E and 2F) and reduced air sac spaces and thickened hypercellular intersaccular septa (Fig 2G and 2H) with an elevated phosphorylation level of Smad1/5/8 (S2D Fig) in lung tissues, confirming the essential role of Fstl1 in lung development.

### Airway smooth muscle malformation in *Fstl1* null lungs

**Fig 3:**
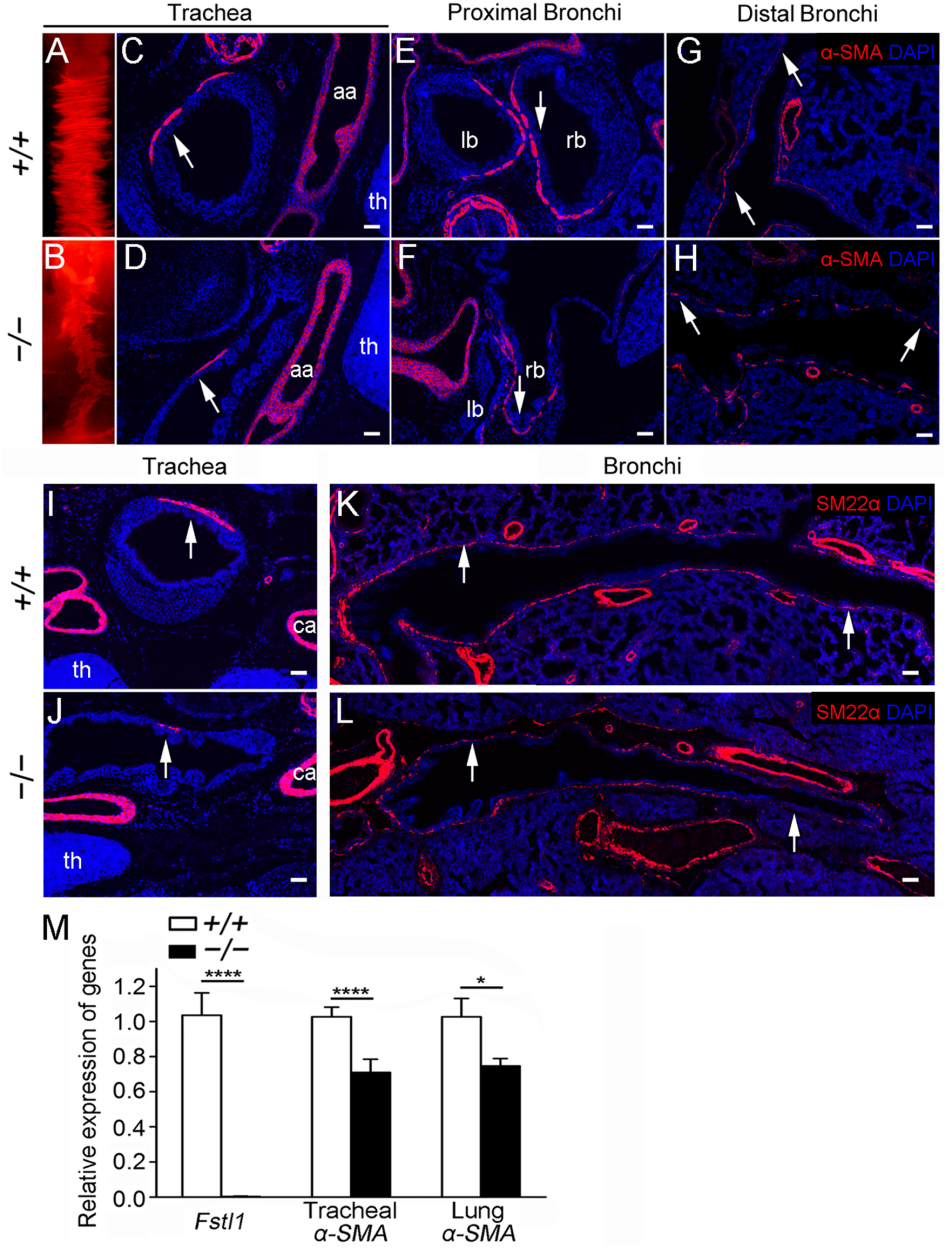
Loss of Fstl1 led to abnormal tracheal and bronchial SM formation in E18.5 embryos. (**A**, **B**) α-SMA whole-mount staining revealed an extremely attenuated α-SMA signal in *Fstl1*^*−/−*^ trachea. Trachea (**C**, **D**), proximal bronchi (**E**, **F**, the sections at the points where the tracheas split into the left and right main bronchi) and distal bronchi (**G**, **H**) sections stained for α-SMA confirmed reduced α-SMA expression (arrows). (**I**, **J**) Trachea sections of similar planes, as indicated by the common carotid artery and thymus, stained for SM22α revealed reduced SM cells in *Fstl1*^*−/−*^ trachea. (**K**, **L**) Stitched images showed airway SM defects from proximal bronchi to distal bronchi in *Fstl1*^*−/−*^ lung. (**M**) qRT-PCR analysis of the expression of *Fstl1* and *α-SMA* in E18.5 tracheas and lungs (n = 5 per group). aa, arch of the aorta, th, thymus, ca, common carotid artery, lb, left main bronchus, rb, right main bronchus. *, P < 0.05; ****, P < 0.0001. Error bars indicate mean ± SEM. Scale bars, A, B, 200 μm; C-L, 50 μm.

To determine the function whereby Fstl1 expression localized in SM cells described earlier (Fig 1B), E18.5 *Fstl1*^*−/−*^ tracheas and lungs were examined for ASM defects. In E18.5, tracheal SM is aligned in the dorsal part of the trachea and connects the endings of the tracheal C-ring cartilage to maintain the shape of trachea during gas exchange [26]. Whole-mount immunofluorescence analysis of α-SMA expression, a marker of SM cells, revealed a significantly smaller and irregular line of SM in *Fstl1*^*−/−*^ trachea (Fig 3B), when compared to that of WT (Fig 3A). Immunofluorescent staining of α-SMA on transverse sections of homozygous tracheas of similar planes, as indicated by the arch of aorta and thymus, confirmed the reduction of α-SMA signal in homozygous tracheas than those of WT (Fig 3C and 3D, arrows). Similar observation was also found in the trachea of our other *Fstl1*^*−/−*^ strain [17] (S3A Fig, arrows). We further examined the SM formation surrounding the proximal and distal bronchi in E18.5 *Fstl1*^*−/−*^ lungs. As expected, α-SMA immunofluorescence analysis of WT lungs showed neatly arranged SM surrounding the proximal (Fig 3E, arrow) and distal bronchial (Fig 3G, arrow) airways, whereas it was interrupted and the α-SMA expression was significantly decreased in *Fstl1*^*−/−*^ lung airways (Fig 3F and 3H, arrows). *Fstl1*^*−/−*^ lung cross-sections of similar planes displayed sporadic α-SMA-expressing cells around the airways, resulting in significant gaps in the sub-epithelial ASM layer. Therefore, the high expression level of Fstl1 in SM cells is essential for ASM formation. *Fstl1* deficiency leads to a severe ASM defect in *Fstl1* null embryos.

To verify this conclusion, we used another mature SM cell marker, SM22α [27], to immunostain sections of similar planes from both WT and *Fstl1*^*−/−*^ tracheas and lungs. As expected, we observed a significant decrease of SM22α immunofluorescent signals in E18.5 *Fstl1*^*−/−*^ trachea (Fig 3J, arrow), as well as in proximal bronchi to distal bronchi from E18.5 *Fstl1*^*−/−*^ lungs (Fig 3L, arrows). Moreover, qRT-PCR analysis confirmed the reduced *α-SMA* mRNA expression in E18.5 *Fstl1*^*−/−*^ tracheas and lungs with removal of main pulmonary arteries (Fig 3M).

### Reduced ASM differentiation in *Fstl1* null lungs

**Fig 4:**
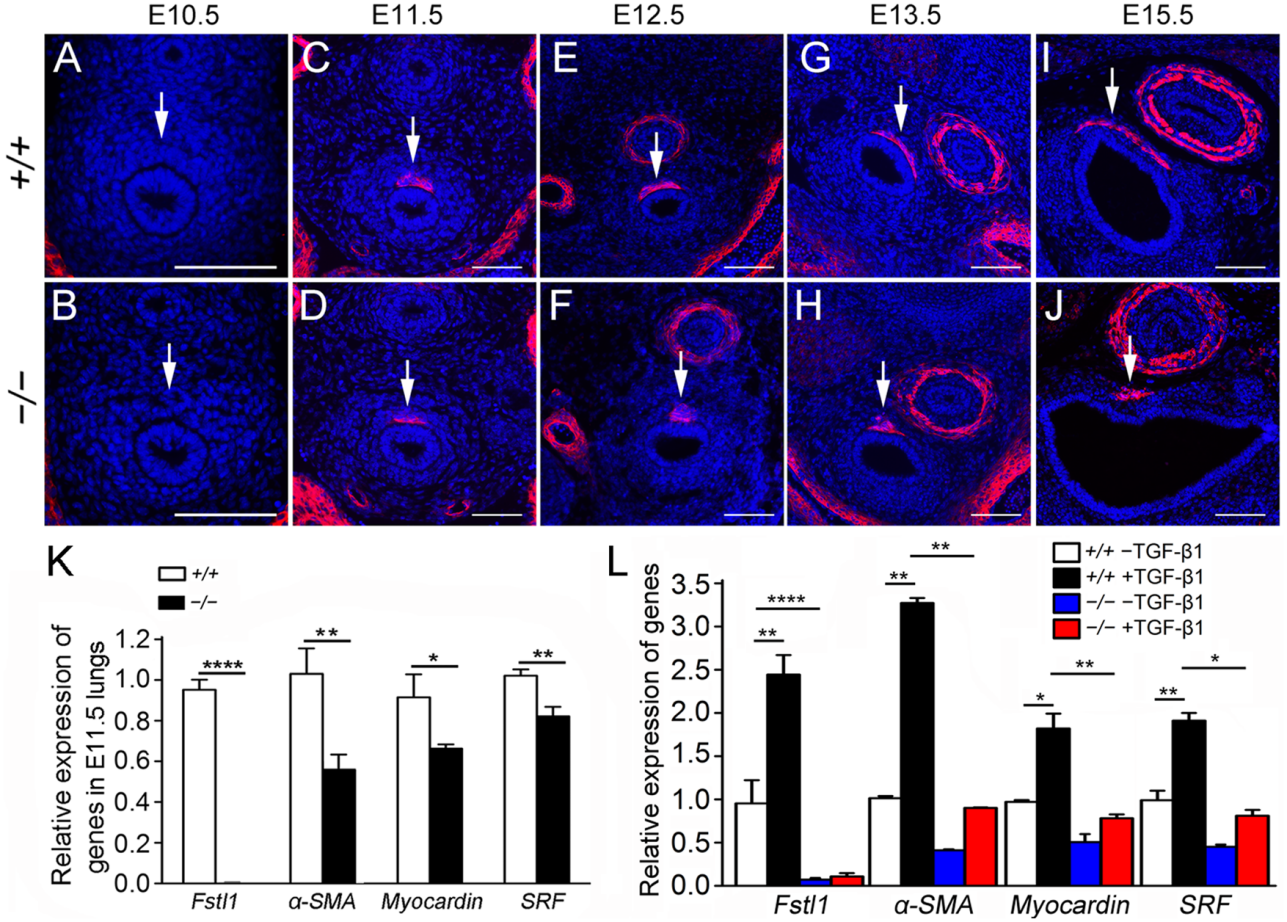
ASM differentiation was significantly reduced in *Fstl1*^*−/−*^ lungs. (**A**, **B**) α-SMA immunostaining of E10.5 trachea sections revealed rare SM cell differentiation in both WT and *Fstl1*^*−/−*^ (arrows). Immunofluorescence staining for α-SMA of E11.5 (**C**, **D**), E12.5 (**E**, **F**), E13.5 (**G**, **H**), E15.5 (**I**, **J**) tracheas showed less SM formation and expansion in *Fstl1*^*−/−*^ tracheas during the early development (arrows). (**K**) qRT-PCR of *Fstl1*, *α-SMA*, *myocardin* and *SRF* expression demonstrated a significant reduction in E11.5 *Fstl1*^*−/−*^ lungs compared to control WT lungs (WT, n = 5, *Fstl1*^*−/−*^, n = 6). (**L**) Loss of Fstl1 inhibited the TGF-β1-induced *α-SMA*, *SRF*, and *myocardin* expression in MEFs. *, P < 0.05; **, P < 0.01; ****, P < 0.0001. Error bars indicate mean ± SEM. Scale bars, 50 μm.

ASM develops from local mesenchymal cells that begin to express SM-specific proteins at the initiation of the pseudoglandular stage (E11.5) and ASM cells are first detected in the trachea [4,26,28]. To determine whether ASM differentiation occurred properly in *Fstl1*^*−/−*^ embryos, α-SMA was used to immunostain sections of E10.5-E15.5 tracheas (Fig 4A–4J). As expected, few α-SMA^+^ cells were detected in sections of E10.5 WT and *Fstl1*^*−/−*^ tracheas (Fig 4A and 4B, arrows). The signals appeared at E11.5 in the dorsal part of both WT and *Fstl1*^*−/−*^ tracheas, then increased and expanded up to E15.5. However, compared to WT, the signal intensity of α-SMA was lower in *Fstl1*^*−/−*^ tracheas and the expansion range was remarkable smaller (Fig 4C–4J, arrows). Thus, our data suggest that Fstl1 deficiency inhibits the differentiation and movement of ASM in lung mesenchyme.

To test our hypothesis, we measured the expression of *α-SMA* and two transcript factors *myocardin* and *SRF*. Myocardin and SRF, critical upstream regulators of myogenesis and in primitive SM cells, are expressed in the developing and matured SM of several tissues including the lung, and transactivate numerous SM differentiating genes including α-SMA [26,29]. As shown in (Fig 4K), mRNA expression levels of *α-SMA*, *myocardin* and *SRF* were significantly decreased in E11.5 *Fstl1*^*−/−*^ lung buds when compared to that of WT, indicating the impaired early myogenic transcriptional program in the lung. Our data suggest that Fstl1 is required for the initiation of the ASM gene program in the developing lung.

TGF-β1-induced differentiation of SM cells via SRF and myocardin in mesenchymal and SM cells was frequently reported [30–33]. We previously reported that Fstl1 addition increased expression of α-SMA via activation of TGF-β-Smad signaling in lung fibroblasts, which suggested a role of Fstl1 in regulating the differentiation of SM cells [16]. MEFs were reported to be highly heterogeneous and exhibit anatomic and developmental variation and serve as multipotent progenitors [34]. To further define the mechanisms of the defects of ASM in *Fstl1*^*−/−*^ tracheas and lungs observed *in vivo* studies, we used an *in vitro* model of TGF-β1-treated mouse fibroblasts to test the effects of Fstl1 on SM cell differentiation. We isolated MEFs from E13.5 WT and *Fstl1*^*−/−*^ embryos and observed that TGF-β1-induced phosphorylation level of Smad2/3 (S3B Fig) and subsequent mRNA expressions of *α-SMA*, *SRF*, and *myocardin* in WT MEFs was remarkably inhibited in *Fstl1*^*−/−*^ MEFs (Fig 4L), indicating that Fstl1 positively regulates α-SMA expression and ASM differentiation via the key transcriptional factors, SRF and myocardin. Collectively, these data suggest that Fstl1 has a important role in ASM formation in lung development.

### Vasculature smooth muscle malformation in *Fstl1* null lungs

E18.5 *Fstl1*^*−/−*^ lungs were also examined for VSM formation. Although the α-SMA immunostaining was comparable in blood vessels of pulmonary arterioles (S3C Fig), we observed a significant hypertrophy of VSM surrounding the pulmonary arteries of E18.5 *Fstl1*^*−/−*^ lungs, as indicated by the thickened α-SMA immunostaining (S3C Fig). The VSM hypertrophy was also observed in the lung of our other *Fstl1*^*−/−*^ strain [17] (S3D Fig). Findings suggest that Fstl1 is important for VSM formation in lung development.

### SM formation in other organs of *Fstl1* null embryos

**Fig 5:**
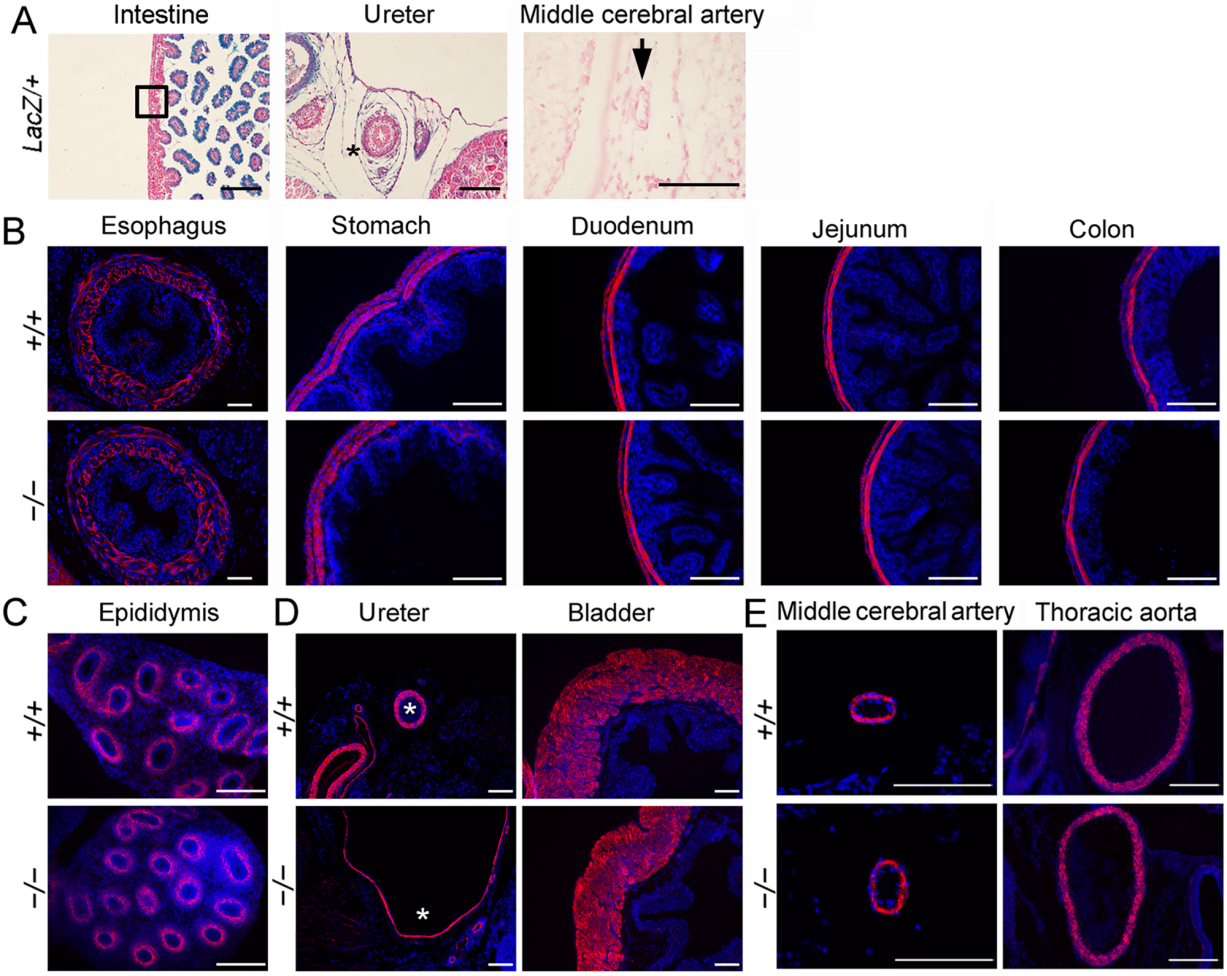
LacZ staining and immunostaining for α-SMA on transverse sections of other organs. (**A**) LacZ staining of intestine, ureter and middle cerebral artery sections. (**B**) Immunostaining for α-SMA on transverse sections of E18.5 esophagus, stomach, duodenum, jejunum and colon. (**C**) α-SMA staining of epididymis showed normal SM differentiation in ductus epididymis. (**D**) α-SMA immunostaining to examinate the formation of SM of ureter and bladder. Asterisks indicate the SM layer of ureter. (**E**) Immunostaining for α-SMA on middle cerebral artery and thoracic aorta sections. Scale bars, 100 μm.

Fstl1 is expressed in ASM and VSM and crucial for ASM and VSM formation in developing lung. We wonder whether Fstl1 is also expressed in the SM cells and important for SM formation in other organs, including digestive system, urinary system and reproductive system. We first used E18.5 *Fstl1*^*LacZ/+*^ embryos to visualize Fstl1 expression in these systems. X-gal staining showed rare LacZ signals in the muscle layer of the intestine (Fig 5A, boxed area) and the middle cerebral artery wall (Fig 5A, black arrow). However, X-gal staining of ureter sections showed a generalized expression of Fstl1 with high levels in the mesenchymal cells surrounding ureteric epithelium (Fig 5A, black asterisk). This is in agreement with the studies in which *Fstl1* expression was low or absent in smooth muscle of developing gut [21] and *Fstl1* mRNA was mainly produced in ureteral mesenchymal cells surrounding ureteric epithelium by *in situ* hybridization [18]. We then immunostained sections of E18.5 *Fstl1*^*−/−*^ embryos with anti-α-SMA antibody. Examination of sections of organs in digestive system, including esophagus, stomach, duodenum, jejunum and colon (Fig 5B), and in reproductive system, such as epididymis (Fig 5C), revealed comparable SM formation in both WT and *Fstl1*^*−/−*^ embryos. In E18.5 urinary system, *Fstl1*^*−/−*^ ureter was enlarged with thinner layers of urothelium and mesenchyme as reported before [18]. Immunostaining analysis displayed thinner ureteral SM in *Fstl1*^*−/−*^ embryos, when compared to their WT littermates (Fig 5D, white asterisks). However, *Fstl1*^*−/−*^ bladder was normal and the SM formation seemed to be normal too (Fig 5D).

We further examined SM formation in systemic vasculature. Immunostaining analysis revealed comparable SM formation in middle cerebral artery and thoracic aorta in both WT and *Fstl1*^*−/−*^ embryos, indicating the normal differentiation of the SM cells in brain vascular network and aorta (Fig 5E).

## Discussion

During embryonic development, SM development is poorly understood. The wide distribution of smooth muscle in the body is matched by patterns of development that differ in different organs [35,36]. In the developing lung, SM is thought to arise from multipotent lung mesenchyme, during which several signaling molecules from both the foregut endoderm and the surrounding mesoderm are known to be necessary. The findings reported here add new insights into our understanding of SM differentiation and demonstrate a crucial role of Fstl1 in mouse lung SM development. We provide data at cellular and animal levels to support a role of Fstl1 on mouse lung airway SM formation. Tissue-specific function of Fstl1 on SM development is also observed.

In the present study, we offered the following lines of evidence that Fstl1 may be involved in early development of airway SM from lung mesenchyme. High level of Fstl1 expression was localized in SM cells of embryonic lung. Targeting deletion of Fstl1 in mice impaired ASM, as reflected in the smaller ASM line in trachea and discontinued ASM surrounding bronchi. The hypoplastic phenotype of airway SM in *Fstl1*^*−/−*^ mice was characterized by the decreases of an early SM differentiation marker (α-SMA) and a mature SM cell marker (SM22α), as well as the reduced myogenic regulatory transcription factors, myocardin and SRF. In cultured MEF cells, TGF-β1-induced SM differentiation in WT cells, as indicated by the increased mRNA expression of α-SMA and activated myogenic regulatory network (*myocardin* and *SRF)*, were remarkably inhibited in *Fstl1*^*−/−*^ MEF cells.

The mosaic of subpopulations present in pulmonary airway and vascular smooth muscle, suggests multiple differentiation pathways, separate lineages or both, however, the mechanisms underlying are largely unknown [1]. Mailleux et al suggests that ASM originates from a pool of Fgf10-positive cells in the distal lung mesenchyme that surrounds epithelium [37,38]. In concert with BMP4 and Fgf9 from endoderm and mesothelium, Fgf10 maintains the SM progenitor in distal lung mesenchyme. Recent evidence has raised the possibility of a mesothelial cells contribution to the VSM lineage in the developing lung [39]. Several key signals, including Shh, Wnt7a, Wnt7b, Nkx2.1 and midkine [4], are known to regulate the differentiation and integrity of lung VSM. Notably, suppression or targeted deletion of Nkx2.1 resulted in abnormal pulmonary VSM development but the bronchial ASM is well formed [40,41]. Midkine transgenic mice showed an increased expression of SM-specific genes in the vascular SM cells, which resulted in the increase in muscularization of small pulmonary arteries [42,43]. But the bronchial SM in the transgenic mice was normal. In our study, mice lack Fstl1 resulted in impaired ASM, whereas the formation of the SM surrounding the pulmonary arterioles was normal. Findings indicate that Fstl1 is a new factor differentially regulating the differentiation of ASM or VSM in developing lung. Interestingly, in contrast to the normal VSM surrounding the pulmonary arterioles, we observed hyperplasia SM in pulmonary artery in *Fstl1*^*−/−*^ mice. This is in agreement with previous report that Fstl1 is induced in human umbilical artery SM cells and inhibits vascular SM cells proliferation in vitro [44]. We hypothesize that the role of Fstl1 on VSM development may be site specific, such that the regulating process in large vessels differs from that in the small vessels.

The tissue-specific role of Fstl1 on SM development was also illustrated in several other organ systems. In ureter, Fstl1 expression was localized in the mesenchyme surrounding the epithelial cells of developing ureteric bud. Targeting deletion resulted in a thinner cell layer of ureteral SM in *Fstl1*^*−/−*^ mice. This is in agreement with the phenotype of thinner cell layer of ureteral SM from Xu group using another line of *Fstl1*^*−/−*^ mice [18]. Thus, Fstl1 expression is essential in maintaining normal SM formation in these systems. However, in developing gut, intestine muscle layer and middle cerebral artery wall, the expression of Fstl1 was almost undetectable in their SM layer using *Fstl1-lacZ* reporter mice. Similar observation was reported using in situ hybridization analysis [21]. Targeting deletion of Fstl1 had less effect on their SM formation in these organs, suggesting that the tissue-specific role of Fstl1 on SM formation is associated with expression pattern during embryonic development.

We have previously reported that Fstl1 antagonized BMP signaling from the basolateral side of the polarized lung epithelial cells in a paracrine manner to regulate the differentiation/maturation of lung epithelial cells [17]. We have shown here that Fstl1 was expressed with a high level in the SM of embryonic lung. Fstl1 deficiency caused severe defects in both ASM and VSM, which indicated an essential role of Fstl1 in the normal formation of ASM and VSM in developing lung. We further demonstrated that Fstl1 regulated the ASM cell differentiation via regulating the critical transcriptional factors myocardin and SRF. The precise mechanisms that Fstl1 regulates ASM and VSM formation, if and how Fstl1 regulates some important signaling pathways, such as BMP, SHH, WNT and FGF, during lung SM development and SM-related lung diseases including pulmonary artery hypertension, are actively pursued in our laboratories. This study and our continuing efforts will provide insights into the mechanisms of the regulation of SM formation in developing lung and into the understanding of the molecular mechanisms of lung SM anomalies in human, and would provide new strategies for new therapeutic developments.

## Supporting information

S1 Fig Generation of *Fstl1-LacZ* reporter and *Fstl1* knockout mice. (**A**) Southern blot analysis of progenies from chimeric mice and 129/Sv, indicating the WT allele (9.7 kb) and mutated allele (6.5 kb), resulting from BstZ171 restriction enzyme digestion. (**B**) Genotyping of heterozygous *Fstl1*^*LacZ/+*^, *Fstl1*^*loxp/+*^, *Fstl1*^*loxp/loxp*^, *Fstl1*^*+/−*^, *Fst1l*^*−/−*^ mice mice and WT control. (**C**) Semi-qRT-PCR analysis of transcriptional levels of *Fstl1* exons. *β-actin* was used as loading control.
(TIF)

S2 Fig Phenotypes of WT and *Fstl1*^*LacZ/LacZ*^ embryos. (**A**) Gross phenotypes of newborn WT and homozygous *Fstl1-lacZ* reporter mice (*Fstl1*^*LacZ/LacZ*^). H&E staining of trachea (**B**) and lung (**C**) sections of E18.5 WT and *Fstl1*^*LacZ/LacZ*^ embryos. (**D**) Phosphorylated Smad1/5/8 in lung tissues from WT and *Fstl1*^*−/−*^ embryos at E18.5. Scale bars, 50 μm.
(TIF)

S3 Fig Abnormal ASM and VSM formation in *Fstl1* knockout lungs. (**A**) α-SMA immunostaining on tracheal sections of E18.5 WT and *Fstl1* exon 2 knockout embryos. (**B**) MEFs were treated with 5 ng/ml TGF-β1 for 30 minutes and protein expression was determined by Western blot. (**C**) α-SMA immunostaining of pulmonary arterioles and arteries in *Fstl1*^*−/−*^ mice lung. High magnification of the boxed areas on the right. (**D**) H&E staining on main pulmonary artery sections of E18.5 WT and *Fstl1* exon 2 knockout lungs. High magnification of the boxed areas on the right. Scale bars, 50 μm.
(TIF)
