# Supplementary figures and images for: Follistatin like-1 (Fstl1) is required for the normal formation of lung airway and vascular smooth muscle at birth
Source: PLoS One. 2017 Jun 2;12(6):e0177899. doi: 10.1371/journal.pone.0177899 (PMC5456059; doi:10.1371/journal.pone.0177899)

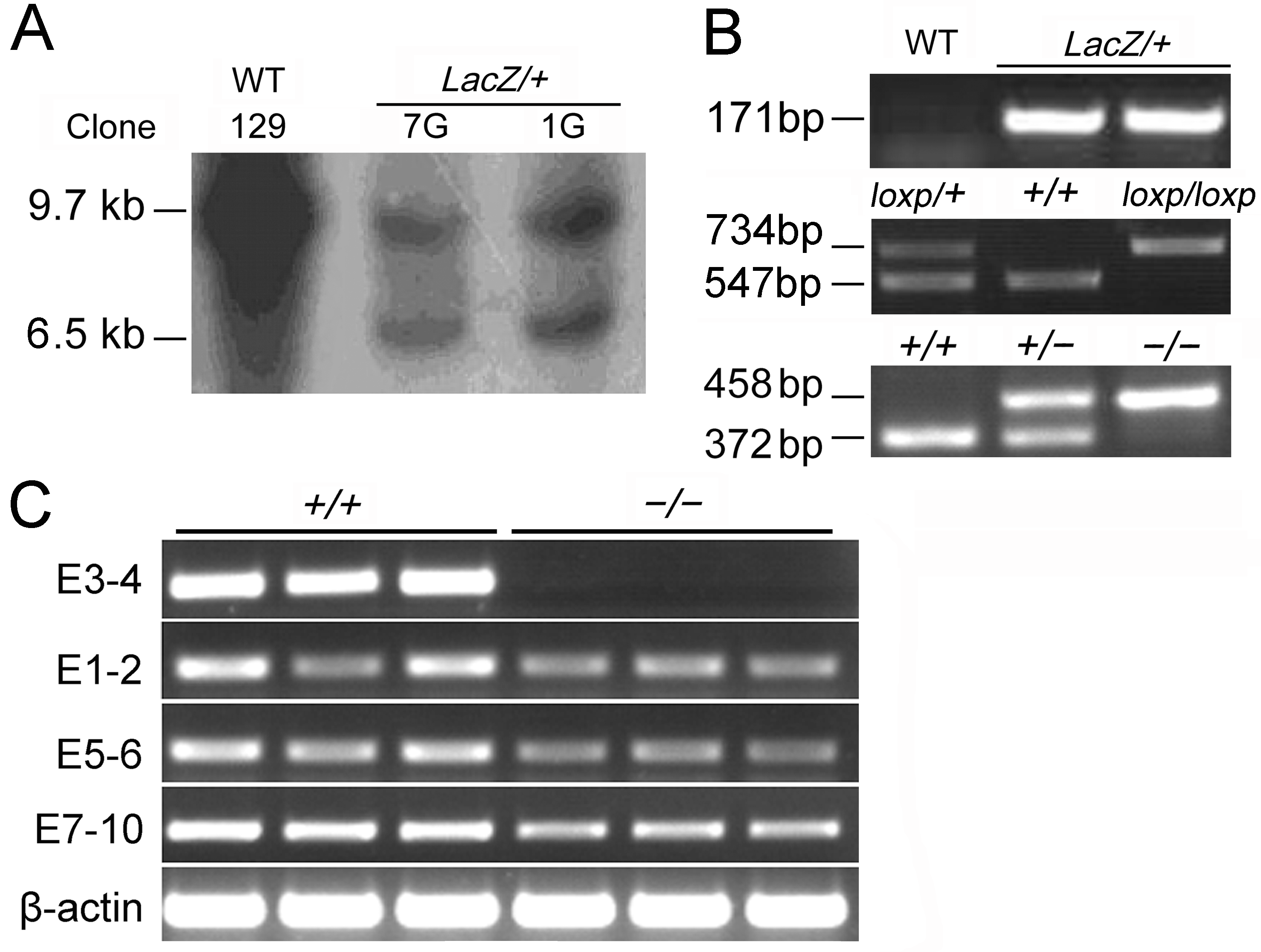

Supplement: S1 Fig — (A) Southern blot analysis of progenies from chimeric mice and 129/Sv, indicating the WT allele (9.7 kb) and mutated allele (6.5 kb), resulting from BstZ171 restriction enzyme digestion. (B) Genotyping of heterozygous Fstl1LacZ/+, Fstl1loxp/+, Fstl1loxp/loxp, Fstl1+/−, Fst1l−/− mice mice and WT control. (C) Semi-qRT-PCR analysis of transcriptional levels of Fstl1 exons. β-actin was used as loading control. (TIF) [file pone.0177899.s001.tif]

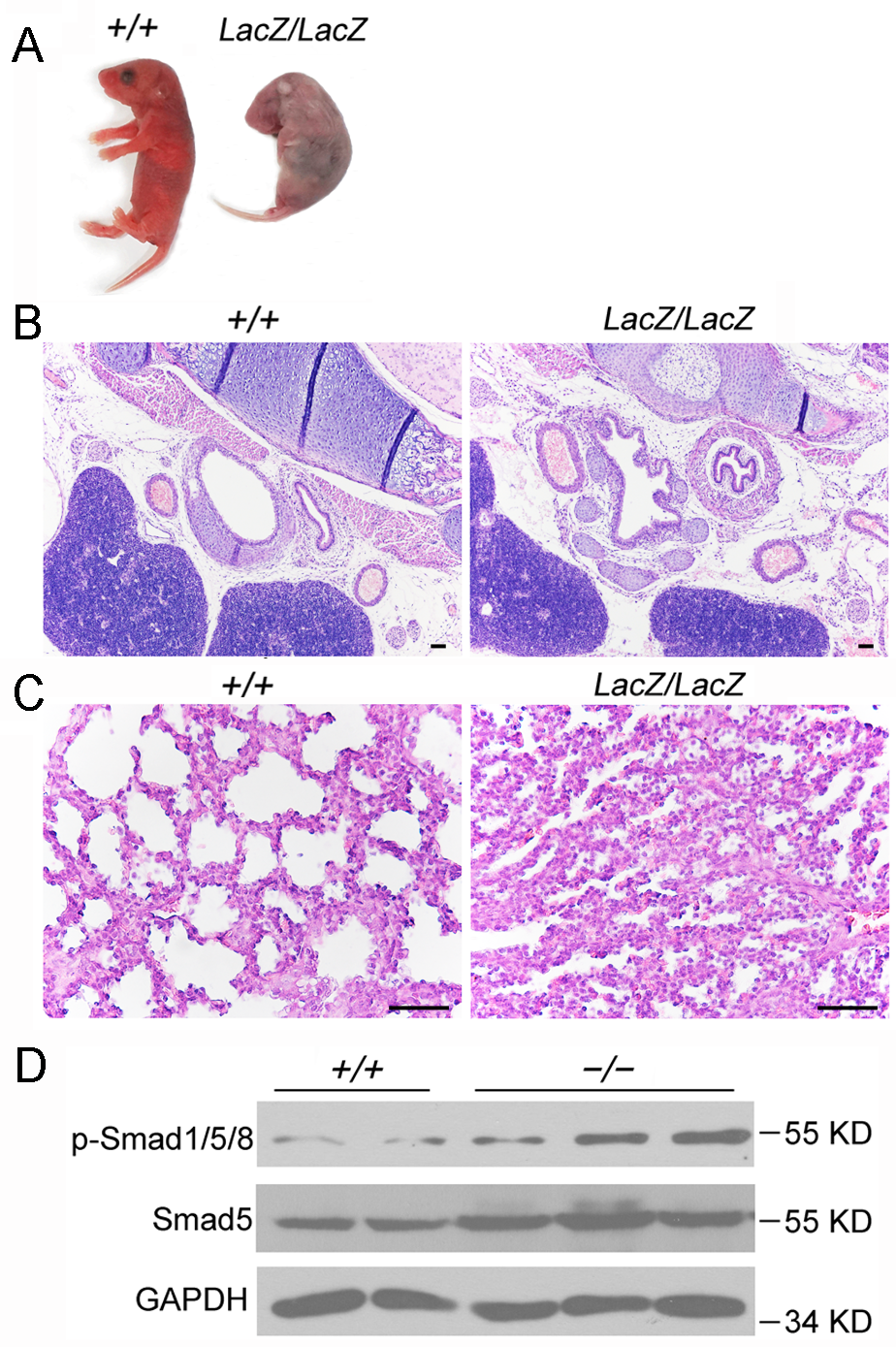

Supplement: S2 Fig — (A) Gross phenotypes of newborn WT and homozygous Fstl1-lacZ reporter mice (Fstl1LacZ/LacZ). H&E staining of trachea (B) and lung (C) sections of E18.5 WT and Fstl1LacZ/LacZ embryos. (D) Phosphorylated Smad1/5/8 in lung tissues from WT and Fstl1−/− embryos at E18.5. Scale bars, 50 μm. (TIF) [file pone.0177899.s002.tif]

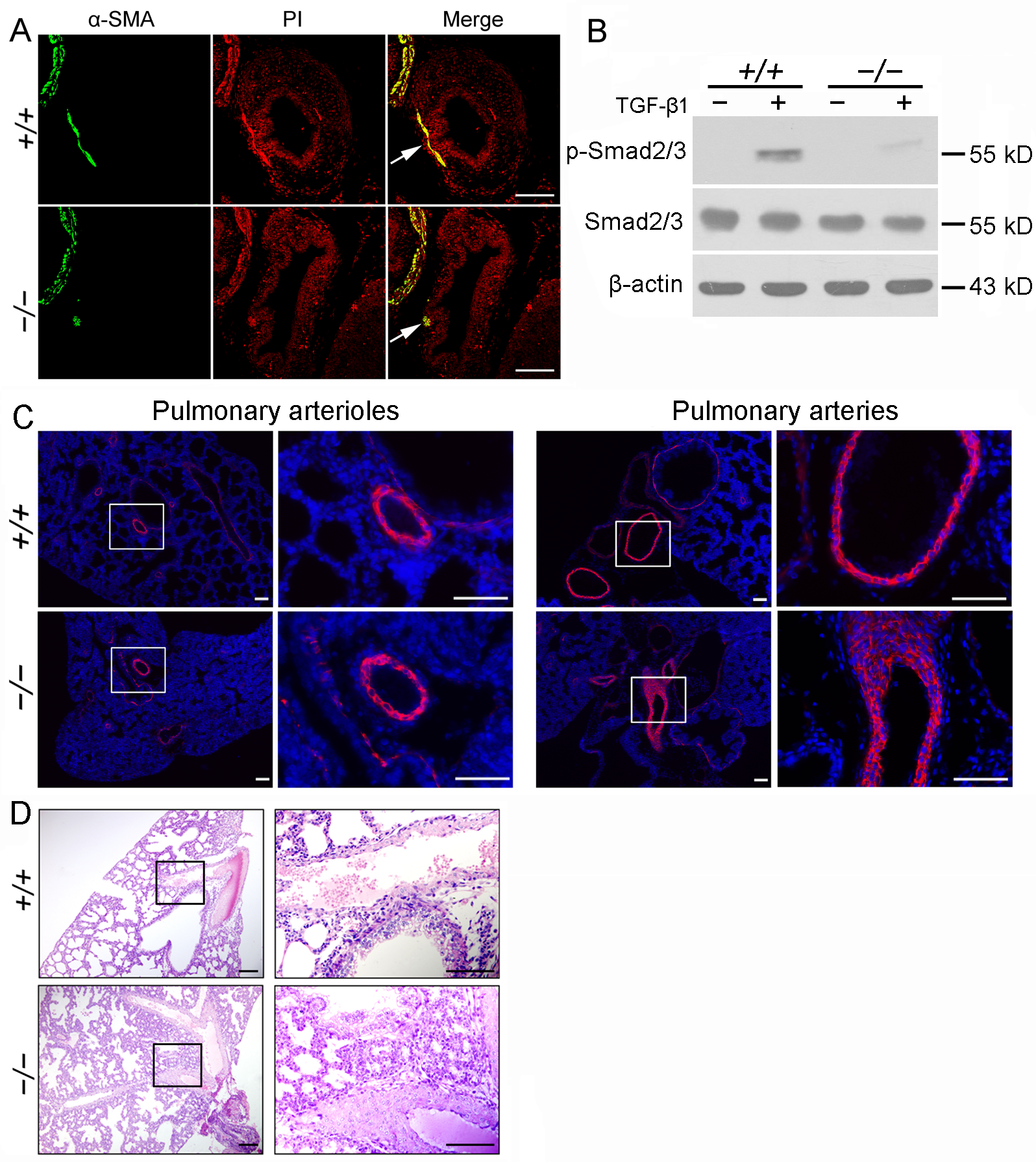

Supplement: S3 Fig — (A) α-SMA immunostaining on tracheal sections of E18.5 WT and Fstl1 exon 2 knockout embryos. (B) MEFs were treated with 5 ng/ml TGF-β1 for 30 minutes and protein expression was determined by Western blot. (C) α-SMA immunostaining of pulmonary arterioles and arteries in Fstl1−/− mice lung. High magnification of the boxed areas on the right. (D) H&E staining on main pulmonary artery sections of E18.5 WT and Fstl1 exon 2 knockout lungs. High magnification of the boxed areas on the right. Scale bars, 50 μm. (TIF) [file pone.0177899.s003.tif]
